# Hypotheses of Spatial Stock Structure in Orange Roughy *Hoplostethus atlanticus* Inferred from Diet, Feeding, Condition, and Reproductive Activity

**DOI:** 10.1371/journal.pone.0026704

**Published:** 2011-11-01

**Authors:** Matthew R. Dunn, Jeffrey S. Forman

**Affiliations:** National Institute of Water and Atmospheric Research Limited, Wellington, New Zealand; Institute of Marine Research, Norway

## Abstract

We evaluate hypotheses for meso-scale spatial structure in an orange roughy (*Hoplostethus atlanticus*) stock using samples collected during research trawl surveys off the east coast of New Zealand. Distance-based linear models and generalised additive models were used to identify the most significant biological, environmental, and temporal predictors of variability in diet, proportion of stomachs containing prey, standardised weight of prey, fish somatic weight, fish total weight, and reproductive activity. The diet was similar to that observed elsewhere, and varied with ontogeny, depth, and surface water temperature. Smaller sized and female orange roughy in warmer bottom water were most likely to contain food. Fish condition and reproductive activity were highest at distances more than 20 km from the summit of the hills. Trawl survey catches indicated greater orange roughy densities in hill strata, suggesting hill habitat was favoured. However, analyses of feeding, condition, and reproductive activity indicated hill fish were not superior, despite fish densities on hills being reduced by fishing which, in principle, should have reduced intra-specific competition for food and other resources. Hypotheses for this result include: (1) fish in relatively poor condition visit hills to feed and regain condition and then leave, or (2) commercial fishing has disturbed feeding aggregations and/or caused habitat damage, making fished hills less productive. Mature orange roughy were observed on both flat and hill habitat during periods outside of spawning, and if this spatial structure was persistent then a proportion of the total spawning stock biomass would remain unavailable to fisheries targeting hills. Orange roughy stock assessments informed only by data from hills may well be misleading.

## Introduction

Orange roughy is a long-lived, low productivity, vulnerable deep-sea fish that has been targeted by industrial deep-sea trawl fisheries worldwide [1]–[3]. The productivity of orange roughy is thought to be one of the lowest of all exploited fishes, a consequence of a longevity that may exceed 100 years, and maturity that may not occur until 20–40 years of age [1], [2]. Catch rates in most orange roughy fisheries declined rapidly following exploitation, and as a result orange roughy fisheries have been closed around Europe, Chile, Southern Africa, Australia and New Zealand. The only large-scale commercial fisheries remaining for orange roughy in 2011 are around New Zealand, with the most recent annual catch being about 9200 t, even though many of the New Zealand stocks have been depleted (biomass fished down to below 20% of initial levels [4]). As of 2011, the largest stock, on the east and south Chatham Rise, has collapsed and is apparently continuing to decline despite catch reductions; the stock on the northwest Chatham Rise was depleted in 2006 and recently most members of the fishing industry have agreed to refrain from fishing there for a few years; stocks around the far north and south of New Zealand appear to have declined substantially in many areas, but are of unknown status; the two stocks off the west and south coasts of the South Island were depleted and closed in 2000 and 2007 respectively; however of these the stock on the Challenger Plateau has been estimated to have recovered and was reopened to commercial fishing, on a small scale, in 2010 [4]. The final stock, on the east coast of the North and South Islands (known as the Mid-East Coast), was expected to be rebuilding after being depleted in the mid-1990s [5], but a new assessment, in 2011, indicated the rebuild had not occurred and the stock remains depleted [4].

In New Zealand, various problems with quantitative stock assessment model assumptions have exacerbated uncertainty in stock status [2], [4], [6]–[8]. One population model assumption being investigated for Mid-East Coast orange roughy concerns spatial structure. Previous models have assumed each stock to be a single, homogenous unit. Spatial structure in the stocks has been observed, however, with juvenile orange roughy found in greatest abundance in relatively shallow water (850–900 m), extending into deeper water as they grow (to about 1500 m), with the largest fish more frequently found on and around hills (<9 km from the summit) [9]–[11]. The year-round association between hills and large aggregations of orange roughy is well known, with non-spawning aggregations assumed to occur primarily because the hills may offer better foraging opportunities [12]–[13]. Almost all orange roughy fisheries are spatially distinct, targeting fish aggregations on and around specific underwater features [12], or on their spawning grounds which sometimes occur over flat ground. The interaction between spatial stock structure and fisheries might, in principle, explain some of the problems encountered in orange roughy stock assessment models. Problems achieving acceptable stock assessment models for deep-water fishes are not confined to orange roughy, but have also occurred for black cardinalfish *Epigonus telescopus* [14], smooth oreo *Pseudocyttus maculates* [15] and black oreo *Allocyttus niger* [16], species which have fisheries, like orange roughy, that target aggregations on and around underwater features such as canyons, ridges, hills and seamounts (all hereafter referred to as “hills”).

Orange roughy have never been tagged to study movements, so in this study we evaluate indirect biological evidence for spatial structuring and connectivity, outside of the spawning season, for two alternative versions of the spatial hypothesis. The first is that, despite there being aggregations on hills, the net benefits of all habitats are the same, such that fish feeding, condition, and the proportion of mature fish that will spawn in a given year is the same everywhere; in this case, the ecology of orange roughy in both habitats is similar and mature and reproductively active fish might be present anywhere. The second is that the ecology of flat and hill habitats differs such that fish outside of the “better” hill habitats are in poorer condition, and so spawn less frequently or not at all; in this case, the spawning biomass would only be fish from the hills, and any non-spawning (resting) adults [8] would predominantly be away from the hills. These two different spatial hypotheses both require ontogenetic habitat shifts and fidelity, as suggested by previous studies [10], [11], but for each the vulnerability of the spawning stock biomass to exploitation could be very different. It should be possible to distinguish between the two hypotheses using indirect biological observations, because if there is no difference in ecology between habitats, then the fish from hill and non-hill (hereafter referred to as “flat”) habitats might show little difference in diet or feeding activity, and should show no difference in condition or the proportion of fish with gonads that had spawned or were developing to spawn that year (hereafter “reproductive activity”). In this study we test the ability of different predictors, including the environmental predictors previously used to describe patterns in fish length structure [11], to describe the variability in orange roughy diet, feeding, fish condition as judged by fish weight, and reproductive activity, using research trawl survey samples collected outside of the spawning season from the Mid-East Coast stock. Although the fishery on the Mid-East Coast stock initially focused on large spawning aggregations on and around specific hills in the north of the region, since the mid-1990s the fishery has predominantly targeted non-spawning aggregations on hills [4]. Whether, and which, environmental predictors are selected, should indicate which of the competing hypotheses is more likely to be true. In completing these analyses we also provide a detailed quantitative description of orange roughy diet, the only similar previous studies being off the west coast of New Zealand on Challenger Plateau [17], and off southeast Australia [18], and discuss the value of different habitats to orange roughy.

## Materials and Methods

### Ethics

This study was exempt from ethical approval by the NIWA Animal Ethics Committee.

### Diet and feeding statistics

**Figure 1:**
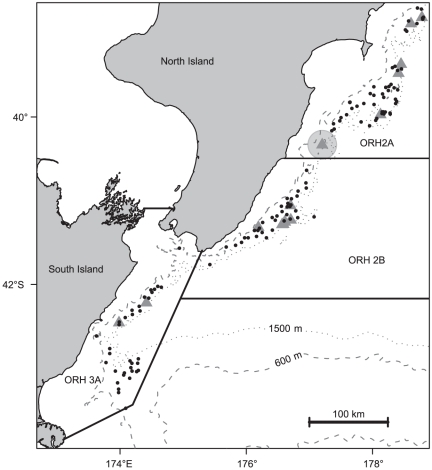
Location of the Mid-East Coast orange roughy stock. Locations where stomach samples were collected (circles), and known hill features (triangles). Dashed line, 600 m isobaths; dotted line, 1500 m isobath. The light grey circle around the hill at the southern border at ORH 2A has a latitudinal radius of 20 km, and is included only for scale.

Biological samples of orange roughy were collected from a stratified random research bottom trawl survey of the Mid-East Coast during March–April 2010 [19] (Figure 1). The sampling area covered 17 358 km^2^, depths between 600 and 1500 m, with strata including flat and sloping continental slope, and hills (features having vertical elevation ≥100 m). The 2010 survey, along with comparable surveys in 1992–94 (Table 1), was timed to take place before any spawning migrations had started, when orange roughy distributions were thought to be stable [19]. Spawning for this stock takes place in June and July [5]. Standardised trawl tows were conducted 24 hours a day, with up to 12 tows completed per day, often across multiple strata. Up to 20 orange roughy were randomly selected for sampling from all tows where they were caught. Selected fish were sampled for standard length (SL, to the nearest mm), total weight (to the nearest 5 g), sex, gonad weight (to the nearest g), and a macroscopic assessment of maturity. The stomach and otoliths were removed for subsequent analysis. At sea, stomachs were sealed by fixing a cable-tie around the oesophagus, then the oesophagus was cut in front of the tie, the intestines cut below the pyloric sphincter, and the stomach removed, labelled, frozen at -20°C and returned to the laboratory. Fish with obviously regurgitated or everted stomachs were not sampled for stomachs.

**Table 1:** Research trawl surveys for the Mid-East Coast orange roughy.

Survey date	Vessel	*n* tows	*n* orange roughy measured
Jun 1986*	*RV James Cook*	4	74
Jun-Jul 1986*	*FV Otago Galliard*	80	1528
Jun-Jul 1987*	*FV Arrow*	76	1270
Oct 1989	*FV Will Watch*	163	2853
Mar-Apr 1992	*RV Tangaroa*	165	2311
Mar-Apr 1993	*RV Tangaroa*	203	3241
Mar-Apr 1994	*RV Tangaroa*	190	3128
Mar-Apr 2010	*RV Tangaroa*	154	3104

The number of tows and fish samples available for analyses of orange roughy condition and reproductive activity. Surveys from 1992 to 2010 were a standardised time series.

*, samples excluded from analyses of reproductive activity. Research surveys were completed using either; *FV*, chartered fishing vessels; or *RV*, dedicated research vessels.

The processing of stomach contents and data analyses followed previous diet analyses for hoki *Macruronus novaezelandiae* [20] and macrourids [21]. Briefly, each stomach was thawed, the wet weight of stomach and contents recorded, the stomach contents removed and rinsed with water, and the wet weight of the empty stomach recorded. Recognisable prey items were then identified. For each prey category, the individual prey items were counted, and the wet weight recorded after removal of surface water by blotting paper.

The weight of the stomach contents as a percentage of total weight (%S) was calculated as %S  =  W/(T – F + E) × 100 where W is the weight of the sorted prey, T is the total fresh fish weight (including full stomach), F is the weight of the full stomach, and E is the weight of the empty stomach; this formulation excludes the weight of fluid and fine material found in the stomach from the statistic [22]. Variability in %S, and the proportion of stomachs containing food, were analysed using a series of generalised additive models (GAMs), as implemented in the mgcv library of the statistical package R [20], [23]. The %S was log transformed and modelled using an identity function and Gaussian error term; the proportion of stomachs containing food was modelled using a logit link and binomial error term. The results of the GAM analyses were marginal tests, fitting each predictor individually, and a final GAM which was built followed the guidelines of Wood & Augustin [23] and Wood [24], with the additional criteria that each final predictor should be significant (p≤0.05) and explain at least 0.1% of additional deviance. The potential predictors of %S and the proportion of stomachs containing food included the fish sex; macroscopic maturity stage, length and weight, and the tow year, month, time of day; mean depth; difference in depth between the start and end position, mean water temperature at the surface; mean water temperature at the bottom; the difference between the two temperatures; the distance to the summit of the nearest feature (any known seamount, hill or knoll (having vertical elevation ≥100 m) identified in the NIWA SEAMOUNTS database [25] with a summit depth ≥ 600 m and ≤ 1500 m; Figure 1); and the survey stratum (hill or flat; hills were originally defined using the locations of commercial fishing tows known to be targeting hill areas [19]). Length, weight, time of day, depth, temperature and distance from hill predictors, were all treated as continuous and fitted in the GAM using cubic splines; other predictors were treated as categorical. Predictor distributions and model residuals were examined to ensure the model fit was adequate; as a result the distance to the summit of the nearest feature, and difference in depth between the start and end position, were both square rooted, and depth was log-transformed. Significant and relevant correlations between predictors are reported in the results. The deep-sea habitat of the Mid-East Coast is poorly known, such that more ecologically pertinent habitat predictors (e.g., primary productivity, presence of biogenic habitat, depth of mesopelagic layers) were not available at the spatial and temporal scales required. Predictors for location, such as longitude, latitude, and region, were not included in the analyses because they are indirect environmental descriptors; i.e., they have no direct significance to the fish.

The unidentifiable prey, parasites found in the stomachs, and prey classified as well digested, were excluded from detailed diet analyses. The contribution of different prey items to the diet was determined by the numerical importance (%N), frequency of occurrence (%F), mass (%W) and percentage index of relative importance (%IRI) [26], [27]. Bootstrap methods, consisting of 1000 replicates of random samples, with replacement, of stomachs from the original data set, stratified by tow, were used to estimate 95% confidence intervals around the dietary statistics [28].

To conduct analyses of diet variability, the prey items were aggregated into taxonomic categories. To assess the adequacy of the samples, the cumulative diversity (Brillouin index of diversity, H) of categorised stomach contents was plotted against the cumulative number of stomachs containing food [29]. The mean and 95% confidence interval were calculated from 1000 curves based upon different random orders of the stomachs. The sample was considered adequate if the mean sample diversity (H) was ≥95% of the asymptotic diversity (H_A_), estimated from a fitted curve of the form H  =  a*n*/(1 + b*n*) [22].

Distance-based linear model (DistLM) analysis in PRIMER v6 [30], [31] was used to identify which of the potential predictors explained most of the variability in diet. Prey weight data were first standardised, which assumed that, within each stomach, weight was a better descriptor of the diet than occurrence or prey frequency, and each stomach was an equally good descriptor of overall diet. The data were then square-root transformed, which reduced the influence of dominant prey, and a dissimilarity matrix calculated using Bray-Curtis distances. The potential predictors were the same as used in the GAM analyses, except year and month were excluded. Significant and relevant correlations between predictors are reported in the results. The most significant predictors were selected using the step-wise selection method, and the Bayesian Information Criterion (BIC) [31]. The results of the DistLM analysis were a marginal test, fitting each predictor individually, and a conditional test, fitting each predictor conditional on the predictor(s) already in the model. To further investigate the effects of the predictors identified from the DistLM analysis, the continuous predictors were binned, with bin boundaries chosen so that the number of observations in each bin was approximately equal. The target number of samples in each bin was sufficiently large to describe >95% of the estimated diversity of the overall diet. The binned data were averaged (mean of normalised proportions of prey species weight), square-root transformed, and then the characteristic prey groups identified with SIMPER (similarity percentages). SIMPER decomposes the average Bray-Curtis dissimilarities between all pairs of samples into percentage contributions from each prey species [30]. The actual mean percentage weight of the prey groups identified by SIMPER was then calculated to show the main differences in diet composition between bins.

### Fish condition

Fish condition was investigated by fitting GAMs to fish weight, thereby assuming that heavier fish of a given length were in better condition [32]. For the 2010 survey, the weight analysed was somatic fish weight, defined as total weight minus gonad weight minus prey weight (weight of the full stomach minus weight of the empty stomach). To determine whether there were temporal trends in fish weight, the analysis was then repeated using all available trawl survey data between 1986 and 2010 (Table 1). Because gonad and prey weight were not available for all surveys, the weight used in this analysis was total fish weight.

The GAMs used an identity function and Gaussian error term, and were fitted in the same way as the analyses of feeding statistics. The potential predictors were the same as used for the feeding statistics, except that in the analysis of 2010 somatic fish weight the time of day (no change in condition was expected over 24 hours) and macroscopic maturity stage (considered a consequence of fish condition, not a cause) were both excluded. In the analysis of 1986–2010 total fish weight, year and month were added, time of day was retained (as weight included prey weight), and stratum (inconsistent across surveys) and temperature (not available) were excluded.

### Reproductive activity

Orange roughy sampled during trawl surveys outside of the spawning season (defined as June and July; i.e., the surveys in 1986 and 1987 were excluded; Table 1) that were classified as the macroscopic maturity stages immature or maturing were grouped as “inactive” (i.e., not reproductively active), and all other stages (mature through ripe, running ripe and spent) were classified as “active” [33]. The samples from June and July were excluded to avoid confounding environmental effects of hills with behaviour preferences for hills as spawning sites. Samples outside of June and July would either be post-spawning, or developing to spawn [33]. Similar to the analyses of feeding statistics, a series of GAMs were used to model the proportion active, using a logit link and binomial error term. The potential predictors for reproductive activity were the same as those used for the analysis of 1986–2010 total fish weight.

## Results

### Feeding statistics

**Figure 2:**
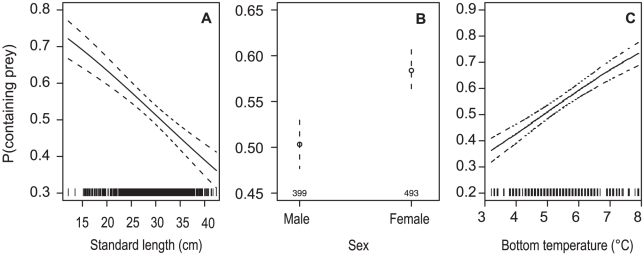
Proportion of orange roughy stomachs containing prey. P(containing prey), generalised additive model predictions (points or solid lines) with 1 SE (dotted lines) for A, orange roughy length; B, sex; and C, bottom temperature, made with all other predictors set to their median (fixed) values. The rug on the x-axis for length and bottom temperature indicates the data points (*n* = 892), for sex *n* is shown above x-axis.

The marginal GAMs on the proportion of stomachs containing prey indicated fish size, depth, time of day, bottom temperature, and temperature difference had the strongest influence; with the habitat predictors, stratum and distance to nearest hill, having no influence (Table 2). The final GAM had the significant predictors length (p≤0.001) + sex (p≤0.05) + bottom temperature (p≤0.001), but explained only 2.8% of the variability. The model suggested a steady decrease in the proportion of stomachs containing prey with increasing fish length, that females more often contained prey, and that the proportion of stomachs containing prey increased with increasing bottom temperature (Fig. 2). There only correlation between a final GAM predictor and other predictors was between bottom temperature and depth (r^2^ = 0.84); other correlations were relatively weak (r^2^≤0.32).

**Table 2:** Percentage deviance explained by predictors in GAM and DistLM analyses.

Predictor	P(containing prey)	%S	Diet composition	Fish somatic weight 2010	Fish total weight 1986–2010	Reproductive activity
Length	1.3**	0.3^NS^	5.6***	96.7***	95.8***	38.8***
Weight	1.3**	0.7^NS^	4.9***	–	–	–
Sex	0.2^NS^	0.01^NS^	0.4^NS^	5.9***	3.9***	19.8***
Gonad stage	0.9*	0.01^NS^	4.5***	59.9***	61.6***	–
Depth	2.3***	0.4^NS^	2.2***	7.9***	3.5***	0.7***
Depth difference	1.5*	1.9^NS^	0.5*	2.1^NS^	2.2***	1.6***
Surface temperature	0.7^NS^	3.8*	1.9***	9.3***	–	–
Bottom temperature	1.5***	0.04^NS^	1.6***	2.0^NS^	–	–
Temperature difference	2.1**	2.1*	2.0***	9.6***	–	–
Stratum	0.1^NS^	0.01^NS^	0.5**	0.01^NS^	–	–
Distance to nearest hill	0.1^NS^	0.1^NS^	0.5*	13.0***	12.7***	5.0***
Time of day	2.2**	0.9^NS^	0.3^NS^	–	0.6***	0.1*
Month	–	–	–	–	5.3***	5.4***
Year	–	–	–	–	5.2***	5.4***

Percentage of deviance explained in marginal tests using Generalised Additive Models for the proportion of orange roughy containing prey, %S, fish weight during the survey in 2010 and surveys between 1986 and 2010, and for diet composition using the DistLM analysis. Approximate significance of predictors: NS, >0.05;

*≤ 0.05;

**≤ 0.01;

***≤ 0.001.

**Figure 3:**
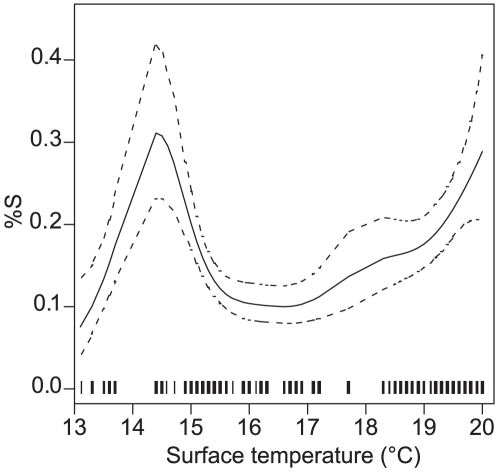
Orange roughy stomach fullness. Stomach fullness (%S) generalised additive model prediction (solid line) with 1 SE (dotted lines) for surface temperature. The rug on the x-axis indicates the data points (*n* = 507).

The marginal GAMs on stomach fullness (%S) indicated only surface temperature and temperature difference had a significant influence (Table 2). The final GAM included only the surface temperature predictor, explaining 3.8% of the deviance. The predicted effect was not a simple trend (Fig. 3), and suggested surface temperature was probably aliasing for something else. Surface temperature was strongly correlated with temperature difference (r^2^ = 0.90).

### Diet

**Figure 4:**
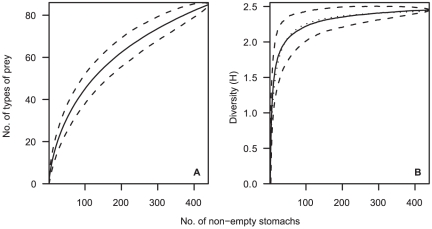
Orange roughy number of prey types and prey diversity with increasing number of stomachs sampled. Panel A, mean cumulative number of prey types identified. Panel B, mean cumulative diversity of prey categories (measured using the Brillouin index of diversity, H). Broken lines indicate the 95% CIs. Dotted line in B is a fitted curve from which asymptotic diversity was estimated. Stomachs containing all unidentifiable or well-digested prey were excluded.

Orange roughy were sampled over a wide spatial area (Fig. 1). Of the 923 specimens examined, 399 (43%) had empty stomachs. Of those containing prey, the analyses of stomach contents led to the identification of 1519 individual prey items in 90 prey groups, having a total weight of 3298 g (Table S1). The number of prey items per stomach ranged between 1 and 78, with 95% of stomachs containing less than 10 prey items, and 50% containing only a single prey item. Prey remains were all unidentifiable or well digested in 80 stomachs, leaving 444 for detailed analyses of diet. The 444 specimens were sampled from a median depth of 956 m (range 671–1386 m), and had a median length of 29.9 cm SL (range 12.1–42.4 cm SL). New types of prey continued to be identified with increasing sample size (Fig. 4A), but the diversity of prey categories reached 95% of the estimated asymptote after 95 stomachs (Fig. 4B), indicating that the sample was large enough to describe the diversity of the diet using the assumed prey categorisation.

The diet of orange roughy was characterised by bathypelagic or mesopelagic bony fishes, crustaceans, and cephalopods (Table S1). Fishes were the most important prey, accounting for 66.8% of the total prey weight. The most important fish prey were lanternfishes (Myctophids; accounting for 15.7% of total prey weight), of which there were at least nine species, followed by small mouth fishes (*Nansenia* spp.; 9.1%), bigscale fishes (Melamphaidae, 6.0%), rattails (Macrourids; 5.2%), and various small fishes such as waryfish (*Scopelosaurus* sp.), daggertooths (Paralepididae), and viperfish (*Chauliodus sloani*). Crustaceans were by far the most common prey, but being relatively small contributed only 21% of the total prey weight. The most important crustacean prey were mysids, shrimps and prawns, predominantly *Pasiphaea* spp. (6.4% of total prey weight), Lophogastridae (3.3%). *Sergestes arcticus* (1.7%), and Boreomysinae (1.4%). *Pasiphaea* aff. *tarda* were less frequent than *P.* aff. *sivado*, but were relatively large and contributed more prey weight. Cephalopods accounted for 11% of total prey weight, and were predominantly bathypelagic squid such as Cranchiidae, including the transparent *Teuthowenia pellucida*, and Onychoteuthidae. Minor prey items included salps and an echinoderm, although the latter seems likely to have been incidental ingestion.

The DistLM analysis indicated significant relationships between diet and several of the predictors (Table 2), with the sequential model having the predictors fish length + depth + surface temperature, together explaining 8.3% of the deviance. There was a significant correlation between depth and bottom temperature (r^2^ = 0.80, p≤0.001), and the deviance explained by a sequential model including length and depth (7.12%) was only marginally better than one using length and bottom temperature (7.11%). The next strongest correlation between final model predictors and other predictors was a weak correlation between bottom temperature and surface temperature (r^2^ = 0.11).

The diet of small orange roughy was characterised by Boreomysinae, *P.* aff. *sivado*, and *S. arcticus* (Table 3). As orange roughy got larger, the diet featured less Boreomysinae and *Pasiphaea* spp., and was characterised more by *S. arcticus*, *Sergia potens*, Oplophoridae, the Onychoteuthidae squids, and lanternfishes *Lampanyctodes* spp. and *Lampanyctus spp*.

**Table 3:** Orange roughy diet by fish length group.

	12.1–25.3	25.4–29.7	29.8–33.3	33.4–42.4
*n*	106	112	111	115
Amphipoda	5.6	3.7	6.3^a^	1.6
Boreomysinae	36.6^c^	18.9^c^	2.0	0.6
*Lampanyctodes* spp.	2.8	10.0^a^	5.2^a^	7.6^a^
*Lampanyctus* spp.	0.7	3.4	8.3^b^	7.8^a^
Lophogastridae	0.0	2.0	7.9^a^	6.8^a^
Onychoteuthidae	0.0	0.0	2.7	4.2^a^
Oplophoridae	1.2	1.0	3.9^a^	10.0^b^
*Pasiphaea* aff. *sivado*	10.5^a^	12.3^b^	3.1	2.8
*Pasiphaea* aff. *tarda*	7.4	8.0^a^	6.5^a^	3.0
Petalophthalmidae	5.0	4.7	3.7^a^	0.9
Salpida	3.2	2.8	3.5^a^	0.9
*Sergestes arcticus*	12.8^a^	11.8^b^	13.1^c^	15.1^c^
*Sergia potens*	0.8	3.4	7.2^a^	7.6^a^

Mean of standardised percent prey weight within the fish length groups (SL in cm), for the prey types together contributing at least 90% of the SIMPER within group similarity for one or more groups. SIMPER percentage contribution to within group similarity:

a 3–10%;

b 10–30%;

c >30%;

no superscript, not identified by SIMPER as characteristic for that group*; n*, sample size.

In relatively shallow water, the diet of orange roughy was characterised by Boreomysinae, *P.* aff. *sivado*, *S. arcticus*, and *Lampanyctodes* spp. (Table 4). As depth increased, the crustacean diet featured less Boreomysinae and *P.* aff *sivado*, and more amphipods, *P.* aff *tarda*, *S. potens*, Lophogastridae, and Oplophoridae, and within the fish diet, *Lampanyctodes* spp. were replaced by *Lampanyctus* spp., Macrouridae and Melamphidae. There was only a weak correlation between orange roughy length and depth (r^2^ = 0.28).

**Table 4:** Orange roughy diet by depth group.

	671–870	873–951	961–1080	1081–1386
*n*	108	114	110	112
Amphipoda	2.6	3.8	4.0	6.7^a^
Boreomysinae	18.6^c^	18.2^c^	10.4^b^	9.4^b^
*Lampanyctodes* spp.	19.8^b^	4.5	0.9	1.0
*Lampanyctus* spp.	1.6	6.8^b^	5.7^a^	6.3^a^
Lophogastridae	1.6	3.2	7.2^a^	4.9^a^
Macrouridae	0.9	0.9	0.1	3.4^a^
Melamphaidae	0.0	0.9	2.6	5.4^a^
Oplophoridae	3.9	0.9	4.8^a^	7.0^b^
*Pasiphaea* aff. *sivado*	16.9^b^	5.8^b^	3.4	2.5^a^
*Pasiphaea* aff. *tarda*	7.1	10.1^b^	4.1^a^	3.4^a^
Petalophthalmidae	2.9	5.2^b^	3.6	2.4
Salpida	2.5	0.8	5.2^a^	1.8
*Sergestes arcticus*	10.1^b^	17.5^c^	18.2^c^	6.9^b^
*Sergia potens*	1.7	3.6	8.1^a^	5.9^a^

Mean of standardised percent prey weight within the depth (m) groups, for the prey types together contributing at least 90% of the SIMPER within group similarity for one or more groups. SIMPER percentage contribution to within group similarity:

a 3–10%;

b 10–30%;

c >30%;

no superscript, not identified by SIMPER as characteristic for that group*; n*, sample size.

The diet in cooler surface water was characterised by Boreomysinae, *Lampanyctus* spp., *P.* aff. *sivado*, and Petalophthalmidae (Table 5). In warmer water, the diet featured less Boreomysinae and more *S. potens*. *Lampanyctodes* spp. replaced *Lampanyctus* spp., and *P.* aff. *tarda* replaced *P.* aff. *sivado*. Amphipoda and Lophogastridae were characteristic of intermediate surface temperatures.

**Table 5:** Orange roughy diet by surface temperature group.

	13.1–15.2	15.3–16.3	16.6–18.9	19.0–20.2
*n*	103	111	113	117
Amphipoda	4.5	5.9^a^	3.7	3.1
Boreomysinae	17.8^c^	14.8^c^	17.4^c^	7.2^a^
*Lampanyctodes* spp.	0.9	0.9	11.8^b^	11.4^b^
*Lampanyctus* spp.	8.7^a^	6.4^a^	4.2	1.7
Lophogastridae	2.9	3.5	8.6^a^	1.8
*Pasiphaea* aff. *sivado*	14.3^b^	11.9^b^	2.1	1.0
*Pasiphaea* aff. *tarda*	6.9^a^	7.0	7.3^a^	7.9^a^
Petalophthalmidae	6.7^a^	7.0^a^	0.0	0.8
*Sergestes arcticus*	11.0^b^	8.9^b^	13.5^b^	19.0^c^
*Sergia potens*	3.0	1.8	7.0^a^	7.3^a^

Mean of standardised percent prey weight within the surface temperature (°C) groups, for the prey types together contributing at least 90% of the SIMPER within group similarity for one or more groups. SIMPER percentage contribution to within group similarity:

a 3–10%;

b 10–30%;

c >30%;

no superscript, not identified by SIMPER as characteristic for that group*; n*, sample size.

### Fish condition

**Figure 5:**
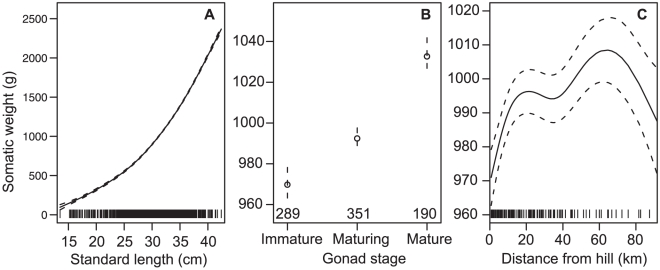
Orange roughy somatic weight from 2010. Generalised additive model predictions (points and solid line) with 1 SE (dotted lines) for A, standard length; B, macroscopic gonad stage; C, distance from the nearest hill. The rug on the x-axis for length and distance from hill indicates the data points (*n* = 830), for gonad stage *n* is shown above x-axis. Immature indicates immature or resting.

The marginal GAMs on fish somatic weight in 2010 indicated length, gonad stage, and distance to the nearest hill had the strongest influence (Table 2). The final GAM had the predictors fish length + gonad stage + distance from the nearest hill, and explained 96.9% of the deviance. The predicted effects showed somatic weight increased with increasing orange roughy length, increased by a small amount (about 6%) with maturity, and varied (by about 4%) with distance from the nearest hill, declining within about 20 km, and further than about 80 km from the nearest hill (Fig. 5).

**Figure 6:**
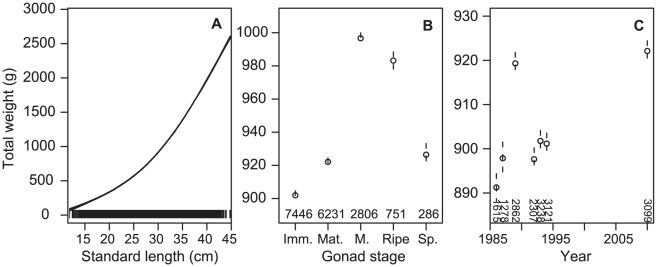
Orange roughy total weight from 1986–2010. Generalised additive model predictions (points and solid line) with 1 SE (dotted lines) for A, standard length; B, macroscopic gonad stage; and C, year. The rug on the x-axis for length indicates the data points (*n* = 17 521), for gonad stage and year *n* is shown above x-axis. Imm., immature or resting; Mat., maturing; Ripe, ripe and running; M., mature; Sp., spent.

The marginal GAMs on total weight for 1986–2010 found all predictors were significant, but fish length, gonad stage, distance from the nearest hill, and year had the greatest influence (Table 2). The final GAM had the predictors fish length + gonad stage + year, and explained 96.1% of the deviance. The predicted effects showed an increase in weight with increasing fish length, and an increase in weight (about 10%) when mature and ripe, consistent with enlarged gonads during these stages, with lowest weight when immature or spent (Fig. 6). The predicted year effect was small (about 3% variability in weight) and suggested inter-annual variability, with no trend (Fig. 6).

### Reproductive activity

**Figure 7:**
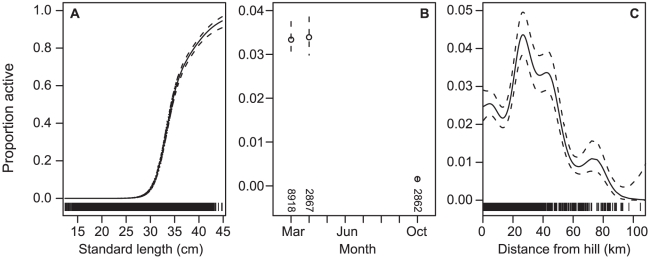
Proportion of orange roughy classified as reproductively active. Using data from 1989–2010, generalised additive model predictions (points and solid line) with 1 SE (dotted lines) for A, fish length; B, month; and C, distance from the nearest hill. The rug on the x-axis for length and distance from hill indicates the data points (*n* = 14 647), for month *n* is shown above the x-axis.

The marginal GAMs on reproductive activity were significant for all predictors (Table 2). The final GAM had the predictors fish length + month + distance from the nearest hill, and explained 46.6% of the deviance. Reproductive activity increased with fish length, was higher in March and April, and was highest at distances of about 25–50 km from the nearest hill (Fig. 7).

## Discussion

Known spatial structuring in the relative abundance of orange roughy, with greater catches on hills than flat habitats, was not easily explained by their ecology, at least for the parameters included in this study. Despite obvious differences in the physical attributes of the hill and flat habitats, and the fact that more fishing for orange roughy had occurred on hills, the ecology of the two habitats was not notably different for orange roughy in terms of amount and type of prey they consumed. Further, orange roughy on hills were not more reproductively active or heavier, and therefore not in better condition than fish caught on flat habitat. These findings apply to the period outside of spawning, and for the duration of our sample collection. Large-scale redistribution of adult fish is known to take place during spawning (as spawning migrations), and because the trawl surveys were temporal ‘snapshots’, it is unknown whether these results would be representative of the entire non-spawning season, or for other years.

We found environmental predictors could explain significant variability in orange roughy diet, feeding statistics, somatic condition and reproductive activity. The variability explained by the available environmental predictors was small however, suggesting that the overall influence of environment on orange roughy biology was small, and might reasonably be ignored when modelling population dynamics. In other words, we believe the spatial structure was not pronounced enough to justify the additional model complexity needed to allow for it, i.e., additional estimable parameters, with the resulting additional uncertainties being propagated into model predictions. As a result, stock assessment models might best assume our first hypothesis; that all mature fish, regardless of their location, can equally contribute to the spawning stock in any given year.

The relatively small influence of environmental predictors may have been because we did not include the most relevant predictors. Alternatively there was genuinely little variability associated with environmental variability. The use of diet and feeding statistics as indicators of habitat variability is the least convincing, primarily because of the sampling of only short time periods [34]. The Mid-East Coast is also a region of complex topography, such that the difference in environmental conditions between hills and flats might be relatively low compared to, for example, a region where a single seamount rises out of a broad surrounding area of flat seabed. While our analyses did not identify strong influences of the environment, they nevertheless give insights into orange roughy biology and hypotheses of spatial structure.

The final analyses for diet and feeding statistics did not select environmental predictors directly associated with the hill versus flat habitat, but fish condition and reproductive activity were estimated to be higher away from the hills. The hills in the analyses all had radii of less than 10 km, so the increase in somatic condition and reproductive activity was outside of the direct influence of the hill. Outside of the spawning season, the reproductively active fish on the flats might not be available to a fishery targeting hills. When most of the fishery catch is taken outside of the spawning season, as is now the case for the Mid-East Coast stock, this interaction of fish and fishery distribution might allow a spatially unavailable or “cryptic” spawning stock biomass to occur.

Aggregations of orange roughy on hills have been the focus for orange roughy commercial fisheries worldwide, even though orange roughy can be ubiquitous in low densities on the continental slope [57]. The predominance of larger orange roughy on hills [9]-[11] indicates that stocks are not homogenous, and might be caused by intra-specific competition for the best habitat. Previous studies have considered the likely value of hill habitats to orange roughy, and concluded hills are more favourable feeding grounds, most likely because of enhanced horizontal flux of mesopelagic prey and extended contact with the mesopelagic layers, and because the seabed is closer and more rugged and so may provide refuges in which to rest or escape from predators [12], [13], [58], [59]. Predictors describing flat versus hill habitats were not selected in our analysis of diet variability, supporting the horizontal prey flux hypothesis rather than hypotheses which presuppose a unique prey fauna available on, or trapped above, hills [58], [60]. Further spatial structure within hill or flat habitats, in response to environmental heterogeneity, does seem quite possible. However, examining this additional spatial structure would require observational data at a scale that does not currently exist for orange roughy.

Orange roughy catch rates in the Mid-East Coast trawl surveys were at least three times higher on hills than flats [19], consistent with hills being a preferred habitat, but our analyses indicated hill fish were not superior. This is probably not a bias in interpretation brought about by orange roughy being easier to catch on hills, as if anything we might expect trawl efficiency to be lower on hills because of the rougher ground. However, trawl independent data, e.g., acoustic surveys, to confirm the higher biomass on hills are not available; acoustic surveys to date have been focused on orange roughy spawning aggregations [5], [8]. At initial biomass levels, the high fish density on hills might result in little net benefit to individuals, but the Mid-East Coast stock has been fished down and catch rates on hills have declined [5], [35] indicating a reduction in local density, and as a result for an individual orange roughy the hill habitat should be better, due to reduced competition for food and resources [12]–[13]. We consider that there are two main hypotheses for hill fish not being superior. First, fish in relatively poor condition may visit hills in order to feed and regain condition, and once this is achieved, they leave. This would imply there are risks of being on hills that outweigh improved feeding opportunities once the fish have gained sufficient condition. Certainly, some hills do attract aggregations of deep-water sharks that are known to predate orange roughy, although it is not clear if orange roughy are eaten live, in the net, or scavenged [36], and fishers report that the presence of orange roughy aggregations on some hills can be intermittent. This “transient fish” hypothesis might be tested by examining temporal changes in the density of orange roughy on hills in relation to fish condition. The second hypothesis is that commercial fishing has disturbed feeding aggregations and/or caused habitat damage, making fished hills a less favourable habitat. Benthic habitat damage by trawling can be substantial, and has been well documented [37], [38]. However, some hill areas have continued to support large orange roughy fisheries despite extensive trawling, suggesting benthic habitat damage may not be that influential [38]. For example the Andes hill complex off eastern New Zealand has remained the centre of the Chatham Rise orange roughy non-spawning fishery despite just over 8800 targeted trawl tows completed over 18 years, in an area less than 18 km across [39]. Disturbance of feeding behaviour by fishing might be substantial however, and fishers often cite recent disturbance as a reason for low catch rates, and try to avoid areas recently fished as a result. This “fishing disturbance” hypothesis could be investigated by examining feeding success (e.g., %S) in relation to time since disturbance, or fish condition in relation to accumulated disturbance.

The diet of orange roughy on the Mid-East Coast was similar to that observed elsewhere, consisting of bentho- and mesopelagic crustaceans, fishes and squid, with crustaceans predominating in the diet of small orange roughy, shifting to fish and some squid as orange roughy got larger [17], [18]. We analysed diet at a more detailed taxonomic level than previous studies, which typically analysed diet at a prey family, order, or class level, and we found some changes in diet with depth and surface water temperature at the prey genus and species level. For example, within the Myctophidae we found mostly *Lampanyctus spp.* in the diet in warm shallow water, and *Lampanyctodes spp.* in deep cold water, and within the natant decapods we found a shift from *P.* aff. *sivado* in shallow water to *P.* aff. *tarda* in deep water. Details of the distribution of the prey species are lacking, so it is unknown whether these changes in diet are meaningful, but it seems reasonable to assume that they may reflect prey availability. Orange roughy appear to be opportunistic predators, within the constraints of their morphology and benthopelagic habitat.

The feeding statistics indicated that smaller sized and female orange roughy in warmer bottom water were most likely to contain food. The decrease in the proportion of orange roughy stomachs containing prey with ontogeny has similarly been found in the deep-sea hoki [20], and may be a ubiquitous pattern if smaller fish feed more frequently because of a higher metabolic rate [40]. The increase in occurrence of prey in warmer water could be related to an increased metabolic rate [41]. Orange roughy extend into deeper and cooler water as they grow [10], and as the occurrence of prey decreased with increasing depth, it appears that larger fish move into deeper water not because of better feeding opportunities, but perhaps because of reduced metabolic costs in the cooler water, or evolutionary benefits such as reduced intraspecific competition or natural mortality [20], [42]–[44]. Sexual dimorphism in orange roughy is not especially pronounced, with females typically growing only a little faster and longer than males [45], therefore the more frequent occurrence of prey in females might be related to the higher energetic requirement of egg production. While the chosen significant predictors did not explain much of the variability in the proportion of orange roughy stomachs containing prey, the predicted effects did seem reasonable. The predicted effect of surface temperature on %S was not reasonable, however, and suggested surface temperature was not the true cause, but was aliasing for some other, possibly spatial, effect.

Although we did not detect a significant circadian pattern in feeding, in accordance with Rosecchi et al. [17], and with there being no significant diel patterns in demersal trawl catch rates [46], Bulman & Koslow [18] found stomach fullness in adult orange roughy peaked during the night. As the main orange roughy prey are mesopelagic and thought to migrate towards surface waters at night, including *Lampanyctodes spp.*, *Lampanyctus spp.*, *S. arcticus*, and *Pasiphaea spp.* [47], [48], night time foraging adult orange roughy would presumably also have to make vertical migrations. In order to explain their observations, while assuming orange roughy had a demersal habit, Bulman & Koslow [18] suggested the orange roughy prey, including *S. arcticus* and *Lampanyctus spp.*, migrated up the slope from deeper water and through the orange roughy depths on their way to and from the surface water at night. However, in principle, dispersed orange roughy might forage both night and day, extending at night some distance into midwater, where they would not be obvious in acoustic surveys because of their low acoustic target strength [49], and where they might not be caught in midwater trawls [10] because of a strong dive-response to disturbance [50]. In order to explain the lack of diel patterns in demersal trawl catch rates, any vertical excursions would probably have to be moderate (perhaps <300 m), and there would have to be a pronounced dive-response to the trawl warps, such that orange roughy were close to the seabed when a demersal trawl net reached them. The observed differences in orange roughy diet with depth could therefore reflect changes in demersal prey distribution and, if we assume that orange roughy foraged within a moderate distance above the seabed, also a pelagic depth stratification of mesopelagic prey. Juvenile orange roughy could be different however, and not make vertical migrations, as their main mysid prey do not migrate (*Boreomysis rostrata* has a constant mean depth of 600 m [51]), and Bulman & Koslow [18] found more fresh food in their stomachs towards the end of the day, suggesting juvenile orange roughy fed most actively during the day when mesopelagic layers were closer to the seabed; this could suggest an ontogenetic difference in orange roughy foraging behaviour.

To analyse fish condition we could have looked at deviations of individual fish from the conventional allometric model of the form Weight  =  a × Length^b^ [52] but preliminary investigations with this model, for the entire data set and by year, found a good fit to most of the data but a consistent positive deviation from the model fit for small (about <20 cm SL) orange roughy. This positive deviation from the conventional growth model could have been interpreted as persistent good condition in small fish. However, it could also simply have been a morphological feature of orange roughy growth. By using the GAM, which allowed more flexibility in fitting the length effect, our analysis removed any potential variability in condition associated with ontogeny, other than that associated with maturity. The analysis of somatic weight indicated an increase in condition associated with the onset of maturity, which in other species has been attributed to fish in better condition being more able to achieve maturity [53]-[55].

Fish maturity is typically modelled as a function only of age or size, but the deviance in reproductive activity explained by fish size, month, and distance from hill in this study (46.6%) suggested it is more complex than that for orange roughy, perhaps because of a proportion of mature fish do not spawn in a given year [8], [56], or perhaps true reproductive activity in orange roughy is difficult to determine. The prediction of higher reproductive activity in March and April, preceding spawning in June and July, is difficult to explain, as it would have to be attributed to the gonad development of maturing fish, which would require the samples to be dominated by first time spawners, or there would have to be substantial gonad atresia in the two months before spawning in June and July. At present, both seem unlikely explanations. Because sample season and year were to some extent confounded, the predicted seasonal effect might have been aliasing for a year effect, which could be a result of inter-annual variability in the proportion not spawning. Alternatively, it might be attributed to confusion between macroscopically spent (active) and immature or maturing (inactive) fish. Similar to somatic condition, reproductive activity increased away from the hills, although then decreased after about 50 km from the summit as opposed to after about 70 km in somatic condition. Orange roughy away from hills outside of the spawning season were in better condition and also more reproductively active, tending to support the hypothesis that fish condition is linked to reproductive activity.

Although hills habitat might have little benefit to individual fish, the influence of available hill habitat on population size through supporting local high fish densities could still be substantial, and areas with more hills may well support a larger stock. Because observations from hills may not be representative of the whole stock, over short time periods (perhaps 3–10 years), orange roughy stock assessments informed by hill biomass trends and demographic data may be misleading about stock size and status. Over longer time periods, the hills may act as fish aggregating devices and maintain catch rates despite a continuing decline in stock biomass, and fishing on hills could even have a depensatory effect by disrupting feeding. To convincingly model these effects and estimate stock size and status in the absence of absolute biomass estimates, stock assessment models need to allow for spatial structure and movement, and will require spatially stratified catch and biomass information.

## Supporting Information

Table S1 **Orange roughy diet composition.** Bold text lines show the point estimates, and 95% confidence intervals estimated by bootstrap resampling, of the percentage frequency of occurrence (%F), percentage weight (%W), percentage number (%N), and percentage Index of Relative Importance (%IRI), for prey grouped at the taxonomic levels used in the multivariate analyses (n = 444). Under each prey group, the normal text lines show the point estimates of the dietary statistics when calculated for all prey types (i.e., at full resolution), with the prey types that could not be allocated to one of the prey groups (so excluded from multivariate analyses) listed at the bottom of the table (n = 524).
(DOC)
