# Supplementary material for: Hypotheses of Spatial Stock Structure in Orange Roughy Hoplostethus atlanticus Inferred from Diet, Feeding, Condition, and Reproductive Activity
Source: PLoS One. 2011 Nov 1;6(11):e26704. doi: 10.1371/journal.pone.0026704 (PMC3206028; doi:10.1371/journal.pone.0026704)
Supplement: Table S1 — Orange roughy diet composition. Bold text lines show the point estimates, and 95% confidence intervals estimated by bootstrap resampling, of the percentage frequency of occurrence (%F), percentage weight (%W), percentage number (%N), and percentage Index of Relative Importance (%IRI), for prey grouped at the taxonomic levels used in the multivariate analyses (n = 444). Under each prey group, the normal text lines show the point estimates of the dietary statistics when calculated for all prey types (i.e., at full resolution), with the prey types that could not be allocated to one of the prey groups (so excluded from multivariate analyses) listed at the bottom of the table (n = 524). (DOC) [file pone.0026704.s001.doc]

**Table S1** Orange roughy diet composition.

|  | **%F** | **%W** | **%N** | **%IRI** |
| --- | --- | --- | --- | --- |
| **CRUSTACEA** |  |  |  |  |
| **Amphipoda** | **7.06 (4.8–9.8)** | **0.17 (0.06–0.3)** | **2.36 (1.52–3.38)** | **0.91 (0.38–1.78)** |
| Amphipoda unidentified | 2.67 | 0.03 | 0.92 | 0.16 |
| *Cyphocaris richardi* | 1.34 | 0.02 | 0.46 | 0.04 |
| *Oediceroides* spp. | 0.57 | 0.01 | 0.20 | 0.01 |
| *Eurythenes gryllus* | 0.19 | 0.05 | 0.07 | 0.01 |
| *Trischizostoma* spp. | 0.76 | 0.02 | 0.26 | 0.01 |
| *Vibilia* sp. | 0.38 | 0.01 | 0.13 | 0.01 |
| **Copepoda** | **0.91 (0.23–1.82)** | **<0.01 (<0.01–0.01)** | **0.30 (0.07–0.61)** | **0.01 (<0.01–0.06)** |
| Copepoda | 0.76 | <0.01 | 0.26 | 0.01 |
| **Cumacea** | **0.23 (0–0.68)** | **<0.01 (0–0.01)** | **0.08 (0–0.24)** | **<0.01 (<0.01–0.01)** |
| Cumacea | 0.19 | <0.01 | 0.07 | <0.01 |
| **Dendrobranchiata and Caridea** |  |  |  |  |
| **Aristeidae** | **1.14 (0.23–2.05)** | **1.10(0.07–2.60)** | **0.38 (0.07–0.75)** | **0.09 (0.01–0.35)** |
| *Aristeus* spp. | 0.95 | 0.9 | 0.33 | 0.07 |
| **Nematocarcinidae** | **0.23 (0–0.68)** | **0.03 (0–0.09)** | **0.08 (0–0.24)** | **<0.01 (0–0.01)** |
| *Nematocarcinus* sp. | 0.19 | 0.02 | 0.07 | <0.01 |
| **Oplophoridae** | **5.47 (3.42–7.75)** | **3.29 (1.92–4.93)** | **1.9 (1.16–2.77)** | **1.45 (0.55–2.92)** |
| *Acanthephyra pelagica* | 1.15 | 0.75 | 0.39 | 0.08 |
| *Acanthephyra quadrispinosa* | 2.67 | 1.67 | 0.99 | 0.45 |
| *Oplophorus novaezeelandiae* | 0.76 | 0.27 | 0.26 | 0.03 |
| ***Pasiphaea* aff. *sivado*** | **11.16 (8.20–14.35)** | **2.68 (1.73–3.93)** | **9.21 (6.29–12.38)** | **6.76 (3.67–10.74)** |
| *Pasiphaea* aff. *sivado* | 9.35 | 2.19 | 7.97 | 6.09 |
| ***Pasiphaea* aff. *tarda*** | **8.66 (6.15–11.39)** | **5.16 (2.91–8.02)** | **3.2 (6.29–12.38)** | **3.69 (1.78–6.51)** |
| *Pasiphaea* aff. *tarda* | 7.25 | 4.22 | 2.76 | 3.24 |
| ***Sergestes arcticus*** | **23.69 (19.59–27.56)** | **2.03 (1.40–2.84)** | **13.77 (10.09–17.91)** | **19.09 (12.62–26.56)** |
| *Sergestes arcticus* | 19.85 | 1.66 | 11.92 | 17.27 |
| ***Sergia potens*** | **5.47 (3.42–7.52)** | **3.95 (2.15–5.95)** | **1.98 (1.14–2.85)** | **1.65 (0.54–3.20)** |
| *Sergia potens* | 4.58 | 3.23 | 1.71 | 1.45 |
| **Mysida** |  |  |  |  |
| **Boreomysinae** | **27.56 (23.46–31.89)** | **1.69 (0.98–2.54)** | **31.96 (24.93–38.54)** | **47.28 (35.42–55.93)** |
| *Boreomysis rostrata* | 17.56 | 0.42 | 18.63 | 21.44 |
| *Boreomysis* type 2 | 8.02 | 0.95 | 8.82 | 5.02 |
| *Boreomysis* type 3 | 0.38 | 0.01 | 0.20 | 0.01 |
| **Mysinae** | **4.33 (2.51–6.38)** | **0.27 (0.04–0.74)** | **8.52 (1.54–19.91)** | **1.94 (0.22–5.81)** |
| *Amblyops* sp. | 0.19 | <0.01 | 0.07 | <0.01 |
| *Erythropini* sp. | 0.38 | 0.15 | 5.13 | 0.13 |
| Mysinae unidentified | 2.86 | 0.07 | 2.11 | 0.40 |
| *Pseudomma* sp. | 0.19 | <0.01 | 0.07 | <0.01 |
| **Petalophthalmidae** | **7.52 (5.01–10.25)** | **0.20 (0.11–0.33)** | **5.10 (3.04–7.56)** | **2.03 (0.87–3.76)** |
| *Petalophthalmus* aff. *armiger* | 0.95 | 0.02 | 0.33 | 0.02 |
| *Petalophthalmus* sp. | 5.34 | 0.14 | 4.08 | 1.45 |
| **Euphausiacea** | **1.37 (0.46–2.51)** | **0.02 (0.01–0.03)** | **0.46 (0.13–0.89)** | **0.03 (0.01–0.12)** |
| Euphausiacea unidentified | 0.95 | 0.01 | 0.33 | 0.02 |
| *Nematoscelis megalops* | 0.19 | <0.01 | 0.07 | <0.01 |
| **Lophogastridae** | **5.24 (3.19–7.29)** | **4.08 (1.62–7.08)** | **1.75 (1.04–2.54)** | **1.56 (0.50–3.30)** |
| *Gnathophausia elegans* | 0.76 | 0.46 | 0.26 | 0.04 |
| *Gnathophausia* zoea | 0.57 | 0.06 | 0.20 | 0.01 |
| *Neognathophausia gigas* | 0.19 | 0.45 | 0.07 | 0.01 |
| *Neognathphausia ingens* | 2.48 | 2.29 | 0.86 | 0.50 |
| *Neognathophausia* spp. | 0.38 | 0.06 | 0.13 | <0.01 |
|  |  |  |  |  |
| **ECHINODERMATA** |  |  |  |  |
| **Echinidea** | **0.23 (0–0.68)** | **0.02 (0–0.08)** | **0.08 (0–0.26)** | **<0.01 (0–0.01)** |
| Echinidea | 0.19 | 0.02 | 0.07 | <0.01 |
|  |  |  |  |  |
| **TUNICATA** |  |  |  |  |
| **Salpida** | **4.33 (2.51–6.38)** | **0.68 (0.30–1.15)** | **2.89 (1.17–5.06)** | **0.79 (0.21–1.81)** |
| Salpida | 1.53 | 0.15 | 1.32 | 0.14 |
| *Soestia zonaria* | 2.29 | 0.40 | 1.18 | 0.23 |
|  |  |  |  |  |
| **CEPHALOPODA** |  |  |  |  |
| **Brachioteuthidae** | **1.82 (0.68–3.19)** | **0.33 (0.01–1.11)** | **0.68 (0.25–1.27)** | **0.09 (0.01–0.32)** |
| *Brachioteuthis picta* | 0.19 | 0.24 | 0.07 | 0.01 |
| *Brachioteuthis* spp. | 1.34 | 0.03 | 0.53 | 0.05 |
| **Chiroteuthidae** | **0.46 (0–1.14)** | **0.03 (0–0.10)** | **0.15 (0–0.40)** | **0.01 (0–0.03)** |
| *Chiroteuthis mega* | 0.38 | 0.03 | 0.13 | <0.01 |
| **Cranchiidae** | **1.37 (0.46–2.51)** | **4.92 (0.13–11.70)** | **0.46 (0.15–0.84)** | **0.37 (0.01–1.31)** |
| *Cranchia scabra* | 0.19 | 0.57 | 0.07 | 0.01 |
| *Galiteuthis* sp. | 0.19 | 0.02 | 0.07 | <0.01 |
| *Taonius* sp. B | 0.19 | 0.08 | 0.07 | <0.01 |
| *Teuthowenia pellucida* | 0.57 | 3.35 | 0.20 | 0.13 |
| **Gonatidae** | **0.23 (0–0.68)** | **0.13 (0–0.44)** | **0.08 (0–0.25)** | **0 (0–0.02)** |
| *Gonatus* sp. | 0.19 | 0.10 | 0.07 | <0.01 |
| **Histioteuthidae** | **0.23 (0–0.68)** | **0.19 (0–0.67)** | **0.08 (0–0.25)** | **<0.01 (0–0.03)** |
| *Histioteuthis* sp. | 0.19 | 0.16 | 0.07 | <0.01 |
| **Mastigoteuthidae** | **0.91 (0.23–1.82)** | **2.51 (0.02–7.50)** | **0.30 (0.07–0.64)** | **0.13 (0.01–0.66)** |
| *Mastigoteuthis agassizii* | 0.76 | 2.05 | 0.26 | 0.11 |
| **Onychoteuthidae** | **2.28 (0.91–3.87)** | **4.83 (1.34–9.44)** | **0.76 (0.31–1.29)** | **0.65 (0.10–1.88)** |
| *Onychoteuthis banksii* | 0.95 | 2.02 | 0.33 | 0.14 |
| *Onykia ingens* | 0.38 | 1.15 | 0.13 | 0.03 |
| *Onychoteuthis* sp.b (i*mber*) | 0.38 | 0.27 | 0.13 | 0.01 |
| *Onykia* sp. | 0.19 | 0.51 | 0.07 | 0.01 |
| **Sepiolidae** | **0.46 (0–1.14)** | **0.04 (0–0.15)** | **0.15 (0–0.41)** | **<0.01 (0–0.03)** |
| *Heteroteuthis serventyi* | 0.38 | 0.04 | 0.13 | <0.01 |
| **Spirulidae** | **0.23 (0–0.68)** | **0.34 (0–1.17)** | **0.08 (0–0.26)** | **<0.01 (0–0.05)** |
| *Spirula spirula* | 0.19 | 0.28 | 0.07 | <0.01 |
|  |  |  |  |  |
| **ACTINOPTERYGII** |  |  |  |  |
| **Epigonidae** | **0.46 (0–1.14)** | **2.59 (0–7.08)** | **0.15 (0–0.41)** | **0.06 (0–0.46)** |
| *Rosenblattia robusta* | 0.38 | 2.12 | 0.13 | 0.06 |
| **Gonostomatidae** | **0.91 (0–1.82)** | **0.83 (0–2.67)** | **0.30 (0–0.66)** | **0.05 (0–0.29)** |
| *Cyclothone* spp. | 0.57 | 0.01 | 0.20 | 0.01 |
| *Gonostomia* sp. | 0.19 | 0.66 | 0.07 | 0.01 |
| **Myctophidae** |  |  |  |  |
| ***Diaphus* sp.** | **0.23 (0–0.68)** | **0.19 (0–0.67)** | **0.08 (0–0.25)** | **<0.01 (0–0.03)** |
| *Diaphus* sp. | 0.19 | 0.16 | 0.07 | <0.01 |
| ***Gymnoscopelus* sp.** | **1.82 (0.68–3.19)** | **1.06 (0.35–1.95)** | **0.68 (0.24–1.25)** | **0.16 (0.02–0.48)** |
| *Gymnoscopelus piabilis* | 1.34 | 0.72 | 0.53 | 0.11 |
| *Gymnoscopelus* sp. | 0.19 | 0.15 | 0.07 | <0.01 |
| ***Hygophum* sp.** | **0.46(0–1.14)** | **0.25 (0–0.66)** | **0.15 (0–0.39)** | **0.01 (0–0.06)** |
| *Hygophum proximum* | 0.38 | 0.21 | 0.13 | 0.01 |
| ***Lampadena* sp.** | **0.23 (0–0.68)** | **1.76 (0–5.71)** | **0.08 (0–0.26)** | **0.02 (0–0.21)** |
| *Lampadena speculigera* | 0.19 | 1.44 | 0.07 | 0.02 |
| ***Lampanyctodes* sp.** | **7.29 (5.01–9.80)** | **1.95 (1.17–3.04)** | **5.94 (3.75–8.83)** | **2.93 (1.24–5.88)** |
| *Lampanyctodes hectoris* | 6.11 | 1.59 | 5.13 | 2.63 |
| ***Lampanyctus* spp.** | **6.15 (4.1–8.66)** | **13.93 (8.41–20.31)** | **2.13 (1.36–3.20)** | **5.04 (2.14–9.47)** |
| *Lampanyctus australis* | 1.91 | 2.67 | 0.66 | 0.41 |
| *Lampanyctus intricarius* | 0.38 | 1.83 | 0.13 | 0.05 |
| *Lampanyctus lepidolychnus* | 0.19 | 0.58 | 0.07 | 0.01 |
| *Lampanyctus macdonaldi* | 0.95 | 1.45 | 0.33 | 0.11 |
| *Lampanyctus* spp. | 1.72 | 4.85 | 0.66 | 0.61 |
| **Macrouridae** | **1.59 (0.68–2.73)** | **6.32 (0.76–13.23)** | **0.53 (0.19–0.95)** | **0.56 (0.03–1.85)** |
| *Caelorinchus* sp. | 0.19 | 0.90 | 0.07 | 0.01 |
| *Coryphaenoides* spp. | 0.38 | 2.27 | 0.13 | 0.06 |
| *Coryphaenoides subserrulatus* | 0.19 | 1.94 | 0.07 | 0.02 |
| Macrouridae unidentified | 0.57 | 0.05 | 0.20 | 0.01 |
| **Melamphaidae** | **2.28 (1.14–3.65)** | **7.33 (1.90–14.53)** | **0.84 (0.36–1.45)** | **0.95 (0.14–2.68)** |
| *Melamphaes microps* | 0.19 | 1.13 | 0.07 | 0.01 |
| *Poromitra capito* | 0.57 | 2.93 | 0.26 | 0.12 |
| *Sio nordenskjoldii* | 1.15 | 1.93 | 0.39 | 0.17 |
| **Microstomatidae** | **1.82 (0.68–3.19)** | **11.13 (4.08–18.85)** | **0.61 (0.24–1.09)** | **1.09 (0.18–3.06)** |
| *Nansenia* spp. | 1.53 | 9.09 | 0.53 | 0.94 |
| **Nomeidae** | **0.23 (0–0.68)** | **3.17 (0–9.93)** | **0.08 (0–0.25)** | **0.04 (0–0.36)** |
| *Cubiceps* sp. | 0.19 | 2.59 | 0.07 | 0.03 |
| **Notosudidae** | **1.37 (0.46–2.51)** | **2.25 (0.43–5.24)** | **0.46 (0.14–0.87)** | **0.19 (0.01–0.73)** |
| *Scopelosaurus* sp. | 1.15 | 1.84 | 0.39 | 0.16 |
| **Paralepididae** | **1.37 (0.46–2.51)** | **1.29 (0.28–2.69)** | **0.53 (0.14–1.03)** | **0.13 (0.01–0.42)** |
| *Lestidiops* sp. | 0.19 | 0.3 | 0.07 | <0.01 |
| *Macroparalepis macrogeneion* | 0.95 | 0.76 | 0.39 | 0.07 |
| **Phosichthyidae** | **0.23 (0–0.68)** | **3.85 (0–12.44)** | **0.08 (0–0.26)** | **0.05 (0–0.47)** |
| *Phosichthys argenteus* | 0.19 | 3.15 | 0.07 | 0.04 |
| **Platytroctidae** | **0.23 (0–0.68)** | **0.65 (0–2.56)** | **0.08 (0–0.26)** | **0.01 (0–0.09)** |
| *Normichthys* sp. | 0.19 | 0.53 | 0.07 | 0.01 |
| **Scopelarchidae** | **0.23 (0–0.91)** | **0.15 (0–0.52)** | **0.08 (0–0.27)** | **<0.01 (0–0.03)** |
| *Scopelarchus* sp. | 0.19 | 0.12 | 0.07 | <0.01 |
| **Stomiidae** | **1.14 (0.23–2.78)** | **2.54 (0.11–5.83)** | **0.38 (0.08–0.81)** | **0.17 (0.01–0.70)** |
| *Chauliodus sloani* | 0.95 | 2.07 | 0.33 | 0.15 |
|  |  |  |  |  |
| **OTHER** |  |  |  |  |
| Cephalopoda unidentified | 2.10 | 0.11 | 0.72 | 0.11 |
| Crustacea unidentified | 2.48 | 0.17 | 0.86 | 0.16 |
| Fish scales | 1.34 | 0.38 | 0.46 | 0.07 |
| Fish unidentified | 21.56 | 16.72 | 7.90 | 34.03 |
| Prawn, mysid or euphausiid unidentified | 4.58 | 0.67 | 1.71 | 0.70 |
| Unidentifiable | 1.15 | 0.03 | 1.12 | 0.08 |

Bold text lines show the point estimates, and 95% confidence intervals estimated by bootstrap resampling, of the percentage frequency of occurrence (%F), percentage weight (%W), percentage number (%N), and percentage Index of Relative Importance (%IRI), for prey grouped at the taxonomic levels used in the multivariate analyses (n = 444). Under each prey group, the normal text lines show the point estimates of the dietary statistics when calculated for all prey types (i.e., at full resolution), with the prey types that could not be allocated to one of the prey groups (so excluded from multivariate analyses) listed at the bottom of the table (n = 524).
